# Rabies Encephalitis in Malaria-Endemic Area, Malawi, Africa

**DOI:** 10.3201/eid1301.060810

**Published:** 2007-01

**Authors:** Macpherson Mallewa, Anthony R. Fooks, Daniel Banda, Patrick Chikungwa, Limangeni Mankhambo, Elizabeth Molyneux, Malcolm E. Molyneux, Tom Solomon

**Affiliations:** * University of Liverpool, Liverpool, United Kingdom; † College of Medicine, Blantyre, Malawi; ‡ Veterinary Laboratories Agency (Weybridge) WHO Collaborating Centre for the Characterisation of Rabies and Rabies-Related Viruses, Surrey, United Kingdom; § Central Veterinary Laboratory, Lilongwe, Malawi

**Keywords:** Rabies, central nervous system infection, Malaria, zoonosis, dispatch

## Abstract

In a malaria-endemic area of Africa, rabies was an important cause of fatal central nervous system infection, responsible for 14 (10.5%) of 133 cases. Four patients had unusual clinical manifestations, and rabies was only diagnosed postmortem. Three (11.5%) of 26 fatal cases were originally attributed to cerebral malaria.

Rabies is a viral infection of the nervous system, caused principally by rabies virus (genus *Lyssavirus*, family *Rhabdoviridae*) and occasionally by other related enzootic viruses (*1**,**2*). After a brief nonspecific febrile prodrome, patients usually manifest either furious (hydrophobic) or paralytic rabies (*3*). The incidence of rabies in many parts of Africa is unknown, but rabies is probably underdiagnosed.

Cerebral malaria is a common cause of death in African children. It is diagnosed clinically in comatose patients with acute *Plasmodium falciparum* infection and no other apparent cause of reduced consciousness (*4*). However, asymptomatic *P. falciparum* infection is common, and other infections must be excluded before coma is attributed to the parasites alone. As part of a prospective clinical study of viral central nervous system (CNS) infections and cerebral malaria in Malawian children, we investigated fatal cases for rabies virus.

## The Study

At the Queen Elizabeth Central Hospital, Blantyre, Malawi (an area hyperendemic for *P. falciparum* malaria), for 3 years beginning March 2002, we enrolled children (2 months to 15 years) with suspected CNS infection into a study. Suspected CNS infection was defined as a fever or history of fever and at least 1 of the following (*5*): reduced level of consciousness (Blantyre coma score [BCS] <4 [*6*] or for children >10 years of age, Glasgow coma score <14); neck stiffness; photophobia; Kernig sign; tense fontanelle; focal neurologic signs; convulsions. Children were excluded if they had a simple febrile convulsion (*7*) or acute bacterial meningitis (cerebrospinal fluid [CSF] leukocyte count >1,000 cells/μL or a positive Gram stain or bacterial culture).

A full history was obtained and detailed examination performed on admission and at least twice daily until discharge or death. Blood was taken for hematocrit determination, examination for asexual forms of *P. falciparum*, full blood count, blood cultures, biochemical screen, and viral serologic tests. On admission, a lumbar puncture was performed, and CSF was taken for cell count; differential cell count; protein and glucose concentrations; Gram stain; bacterial culture; viral PCR; and viral culture.

Hydrophobia (defined as phobic generalized spasms and inspiratory spasms against a closed glottis in response to the offer of a glass of water) or aerophobia (a similar response to blowing air across the cheek) were defined clinically as rabies encephalitis. Children with a CSF count of 5–1,000 leukocytes/μL or features of septicemia were treated with broad-spectrum antimicrobial agents. Children with malaria parasitemia were treated with parenteral quinine. For fatal cases, permission was sought for autopsy or supraorbital needle biopsy. Samples of human brain tissue were analyzed for rabies virus by using the fluorescent antibody test (*8*), the rabies tissue culture inoculation test, and the mouse inoculation test (*9*). We used reverse transcriptase–PCR to produce sequence data for phylogenetic analysis (*10*). The study was approved by ethics committees in Blantyre and Liverpool, and signed informed consent was obtained from patients’ relatives.

During the 3 years, 1,183 children with suspected CNS infections were assessed; 394 with bacterial meningitis or simple febrile convulsions were excluded, which left 789 children in our study. Rabies encephalitis was diagnosed in 10 children (1.3%) on the basis of history of exposure to a rabid animal and initial clinical manifestations (Table). These included 2 children with *P. falciparum* parasitemia. A total of 779 children had no clinical features of furious rabies at the time of admission; 487 of these children had malaria parasitemia, including 341 with a BCS <2/5 who met the case definition of cerebral malaria. In total, 133 (16.9%) of 789 children died, including 58 (17%) of those with a diagnosis of cerebral malaria and all 10 children who had clinical features of rabies. Consent to perform autopsies or postmortem examination of needle-aspirate samples was requested from the relatives of 82 of the 133 children who died. Twenty-nine (35.4% of those requested) gave consent; 23 for autopsy and 6 for needle samples. This number included 26 children with a diagnosis of cerebral malaria, 2 with a clinical diagnosis of rabies, and 1 with suspected meningitis. Six of these 29 patients who died were positive for rabies virus, including the 2 with typical rabies manifestations (patients 9 and 10), 1 diagnosed with meningitis (patient 12), and 3 who had had a diagnosis of cerebral malaria (patients 5, 6, and 11). Thus, overall 14 (10.5%) of the 133 fatal cases were rabies: 10 diagnosed clinically and 4 that were not diagnosed until postmortem material was studied virologically. Three (11.5%) of 26 patients who died with diagnoses of cerebral malaria and for whom postmortem material was examined actually had rabies encephalitis. Although the clinical manifestations of these 3 children were indistinguishable from those of children with cerebral malaria, histologic examination showed that none of these 3 patients had sequestration of parasitized erythrocytes in cerebral tissue (the pathologic hallmark of cerebral malaria). None of these children, nor the 1 with a diagnosis of meningitis, had hydrophobia or aerophobia, which are characteristic of furious rabies; nor did they have limb pain or paresthesia, which are often reported. The disease progression did have unusual features, however. Patient 6 became comatose, flaccid, and areflexic. Patient 11 had periodic episodes of limb shaking; an electroencephalogram during these events showed generalized slow waves but no seizure activity. Patient 12 behaved oddly when a bag of intravenous fluid was set up, but he was not hydrophobic and readily drank water. None of these 4 children had an obvious history of exposure to a rabid animal, although detailed questioning after admission showed possible exposures (Table).

**Table:** Clinical and diagnostic features of 14 patients for whom the ultimate diagnosis was rabies encephalitis*

Patient no (sex/age,y)	Clinical features	History of animal exposure	Admission coma score†	Malaria slide‡	Clinical diagnosis	Time to death	Postmortem positive results
1 (F/13)	Fever and confusion for 2 d; convulsions, hypersalivation, hydrophobia, aerophobia	Uncertain	13/15	Neg	Rabies	24 h	ND
2 (M/13)	Hallucinations, confusion, for 2 d; thought had "been bewitched"; pyrexia, neck stiffness, drooling hydrophobia, aerophobia	Possible dog bite 6 mo earlier	12/15	Neg	Rabies	12 h	ND
3 (M/6)	Fever for 2 d, convulsions for 1 d; agitated, hydrophobia, aerophobia	Dog bite 3 mo earlier	3/5	Pos (1+)	Rabies	4 d	ND
4 (M/7)	Fever for 2 d, confusion, drooling, hydrophobia, aerophobia	Dog bite 3 mo earlier	4/5	Neg	Rabies	24 h	ND
5 (M/8)	Fever for 2 d, convulsions; confused; rapid deterioration	None§	4/5	Pos (2+)	Cerebral malaria	3 d	FAT, PCR, MIT
6 (M/7)	Headache, fever for 3 d, weak, confused; mild neck stiffness, reduced tone, reflexes; CSF 8 leukocytes /mm^3^, protein 40 mg/dL, glucose 4.8 mmol/L; venous glucose 5.5 mmol/L; deteriorated over 10 d	Cat scratch 3 mo earlier	2/5	Pos (1+)	Cerebral malaria	10 d	FAT,¶ PCR, MIT
7 (M/6)	Fever for 1 d, convulsions; neck stiffness, hydrophobia, aerophobia	Dog bite 2 mo earlier	3/5	Pos (2+)	Rabies	<6 h	ND
8 (M/13)	Restlessness, hypersalivation, hematemesis for 1 d; confused, hydrophobia, aerophobia	Dog bite 3 mo earlier	13/15	Neg	Rabies	<6 h	ND
9 (F/11)	Fever, restlessness for 1 d; agitated, hydrophobia, aerophobia	Dog bite, 1 mo earlier	14/15	Neg	Rabies	4 d	FAT, PCR, MIT
10 (M/7)	Fever confusion for 1 d, hallucination, “bewitched”, hypersalivation, confusion, hydrophobia, aerophobia	Dog bite 2 mo earlier	1/5	Neg	Rabies	24 h	FAT, PCR, RTCIT
11 (F/6)	Fever convulsions for 1 d; status epilepticus, hypotonia, areflexia developed; diffuse slow waves on EEG	None§	1/5	Pos (2+)	Cerebral malaria	<6 h	PCR
12 (M/12)	Fell off bike, head injury, no loss of consciousness; ataxia and confusion developed; neck stiffness, fever; CSF 65 leukocytes/mm^3^ (70% PMN cells) protein 30 mg/dL, glucose 4.2 mmol/L	None§	14/15	Neg	Meningitis	3 d	FAT, PCR, MIT
13 (M/7)	Fever for 2 d, convulsion, reduced conscious; agitated, convulsions, hydrophobia, aerophobia	Dog bite 6 wk earlier	2/5	Neg	Rabies	24 h	ND
14 (M/6)	Fever for 2 d, vomiting 1 d, no convulsions; confused, hydrophobia, aerophobia	Dog bite 2 mo earlier	4/5	Neg	Rabies	24 h	ND

*Neg, negative; Pos, positive; ND, not done; FAT, fluorescent antibody test; PCR, reverse transcriptase–PCR; MIT, mouse inoculation test; CSF, cerebrospinal fluid; RTCIT, rabies tissue culture inoculation test; EEG, electroencephalogram; PMN, polymorphonuclear. †Glasgow coma score /15 or Blantyre coma score /5. ‡*Plasmodium falciparum* parasitemia, graded according to the number of parasitized red blood cells per high-powered field (HPF) 1+= 1–100/100 HPF, 2+ = 1–9/10 HPF, 3+ = 1–9 per field, 4+ = >10/HPF) (*11*). §For patients 5, 11, and 12, a possible exposure history was elicited after the diagnosis of rabies encephalitis became apparent. Patient 5 had been scratched by a dog 6 wks earlier, but it had not appeared rabid; patient 11 had been bitten by a neighbor’s dog 6 mo before admission, although this animal had been vaccinated against rabies, and remained well; patient 12 had been bitten by a neighbor’s dog 4 mo earlier, the dog remained well, although another dog had died after apparently “choking on a rat” (rabid dogs often appear to have something stuck in their throat). ¶Patient 6 CSF was negative for rabies virus by FAT at the Veterinary Laboratories Agency Weybridge but positive when tested in Malawi.

**Figure F1:**
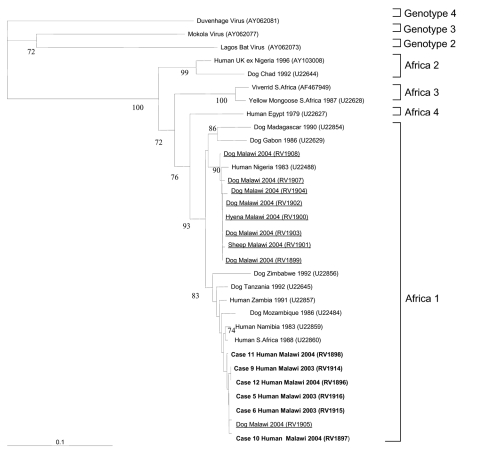
Phylogenetic tree, based on 400 nucleotides of the nucleoprotein gene (*10*), showing the relationship between rabies virus isolates in this study from humans (in **boldface**) and animals (underlined), and other representative isolates; GenBank accession nos. are in brackets. Bootstrap values >70% are considered significant and are included within the figure. The Africa 1, 2, 3, and 4 genotypes are within rabies virus genotype 1 (*13*).

To determine whether the virus isolates in our study were genotype 1 rabies virus, representative of viruses circulating locally, or from other lyssavirus genotypes that also circulate in Africa (e.g., Lagos bat virus genotype 2 or Duvenhage virus genotype 4), we performed a phylogenetic analysis. This analysis included our clinical isolates, rabies viruses we isolated from rabid animals during the course of the study, and other representative strains. We generated consensus trees and bootstrap values as described previously (*12*) and visualized phylogenetic trees with Treeview version 3.2 (Figure). [Fig F1]This analysis showed that all of our clinical isolates were African genotype 1 rabies viruses and were closely related to a virus isolated from a rabid dog during the study.

## Conclusions

This study showed that rabies encephalitis is an important cause of death among children in Malawi. Overall 14 (10.5%) of 133 children who died from suspected CNS infections had rabies. For 10 of these children with hydrophobia, aerophobia, or both, rabies was diagnosed clinically (2 of the cases were also confirmed virologically), but rabies in 4 children with no hydrophobia or aerophobia was not diagnosed until brain material was examined. In 3 of these 4 children, cerebral malaria had been diagnosed. Hypotonia and areflexia developed in 2 children, which might have been a clue that rabies was involved, but these conditions have also been reported in cerebral malaria (*14*). Whether coinfection with malaria parasites might have affected the initial clinical manifestations of rabies is not certain. Overall ≈11% of deaths initially attributed to cerebral malaria were actually due to rabies virus.

Ours was a hospital-based study. The number of rabies cases in the community is likely to be much higher. In some parts of Africa, up to 100 rabies cases are estimated to occur for each 1 officially reported (*15*). Although rabies is a notifiable disease in Malawi, national reporting is hindered by difficulties with the system and the lack of diagnostic facilities. Rabies is preventable by treatment with rabies vaccine after exposure to the virus, and in Malawi several hundred to several thousand people are treated annually with purified chick embryo vaccine. However, in many areas the vaccine is not available, and it was not given to any of our patients, even though several did seek treatment after a dog bite.

In summary, rabies is an important cause of death in children in Malawi, including some for whom cerebral malaria had been diagnosed. Although these patients had unusual disease progression for cerebral malaria, only by examining postmortem material was the diagnosis of rabies made. Rabies virus should be included in the list of pathogens to consider before diagnosing cerebral malaria, especially in fatal cases with hypotonia and areflexia. Attempts should be made to get tissue for diagnosis, e.g., a nuchal skin biopsy, saliva, or CSF antemortem, or a brain sample postmortem.
